# Dejà Vu

**DOI:** 10.3201/eid0907.AD0907

**Published:** 2003-07

**Authors:** Boghos L. Artinian

“I must have made this molecule before; It is familiar to the core!” Said a yeast cell emerging from mitosis, With no experience yet in synthesis. Then, guided by a transmigrant human gene, It assembled that “alien” protein.

